# Changes in the Sensitivity to Language-Specific Orthographic Patterns With Age

**DOI:** 10.3389/fpsyg.2020.01691

**Published:** 2020-07-14

**Authors:** Jon Andoni Duñabeitia, María Borragán, Angela de Bruin, Aina Casaponsa

**Affiliations:** ^1^Centro de Ciencia Cognitiva (C3), Universidad Nebrija, Madrid, Spain; ^2^Department of Language and Culture, The Arctic University of Norway, Tromsø, Norway; ^3^Basque Center on Cognition, Brain and Language, San Sebastian, Spain; ^4^Department of Psychology, University of York, York, United Kingdom; ^5^Department of Linguistics and English Language, Lancaster University, Lancaster, United Kingdom

**Keywords:** orthotactics, orthographic patterns, language-specific orthography, orthographic markedness, aging, reading development

## Abstract

How do bilingual readers of languages that have similar scripts identify a language switch? Recent behavioral and electroencephalographic results suggest that they rely on orthotactic cues to recognize the language of the words they read in ambiguous contexts. Previous research has shown that marked words with language-specific letter sequences (i.e., letter sequences that are illegal in one of the two languages) are recognized more easily and faster than unmarked words. The aim of this study was to investigate sensitivity to markedness throughout childhood and early adulthood by using a speeded language decision task with words and pseudowords. A large group of Spanish-Basque bilinguals of different ages (children, preteenagers, teenagers and adults) was tested. Results showed a markedness effect in the second language across all age groups that changed with age. However, sensitivity to markedness in the native language was negligible. We conclude that sensitivity to orthotactics does not follow parallel developmental trend in the first and second language.

## Introduction

How do bilingual readers identify a language switch? In most bilingual environments, readers can find different cues that help them to recognize a language and access word meaning. Languages with different alphabets (e.g., Greek and Spanish) offer an extreme example: the dissimilar scripts themselves provide enough information to easily differentiate between languages. However, this is not the case for many language pairs. For instance, Italian and Spanish are typologically very similar and share the same alphabet. Thus, readers have difficulties in determining the language of each individual word. Research on visual word recognition with same-script language combinations may help identify what characteristics of such words help with bilingual language selection and recognition.

Orthotactics, the patterns of grapheme combinations in written words, are an important aspect of words, and they are learned by extracting orthographic regularities (Conway et al., 2010; Krogh et al., 2013). Previous research provides evidence for individual sensitivity to the regularity of these letter patterns after little exposure to printed words (Chetail and Content, 2017). In particular, sensitivity increases for letter combinations that belong to an individual’s own language (e.g., higher appearance in the language; Miller et al., 1954), specifically when words include high frequency bigrams (Owsowitz, 1963). Hence, it seems plausible that bilinguals could rely on orthotactic rules as a strategy to differentiate between the languages they know if these share the same alphabet.

Previous research on bilinguals who speak languages that share an alphabet has shown that adults recognize sub-lexical orthographic cues embedded in words very quickly (Vaid and Frenck-Mestre, 2002; Lemhöfer and Radach, 2009; Van Kesteren et al., 2012). For instance, Casaponsa et al. (2014) conducted a study to investigate the sensitivity to orthographic markedness in Spanish-Basque bilinguals. Those languages share the same alphabet but have orthotactically distinct features, such as the bigram “tx,” a letter sequence that exists in Basque but not in Spanish. The task, a speeded language recognition task, consisted of deciding whether items belonged to the participants’ first language (L1) or second language (L2). Both marked words (i.e., words containing bigrams that are legal in only of one of the two languages) and unmarked words (i.e., words containing only bigrams that are legal in both languages) were presented. Results showed that adults were faster at identifying the language of marked words than unmarked words. These results were observed regardless of language proficiency levels (Casaponsa et al., 2014). Interestingly, adult Spanish monolinguals with no prior knowledge of Basque were also tested, and they also showed a markedness effect for Basque-marked words, demonstrating that adults are sensitive to marked language patterns that deviate from their native orthotactic regularities, even when they do not know the language.

A wealth of evidence supports the notion that word recognition in bilinguals is mediated by cross-language lexical activation, even when bilinguals are set in a seemingly monolingual language context (e.g., Van Heuven et al., 1998; Dijkstra et al., 2000; Thierry and Wu, 2007; Midgley et al., 2008; Grossi et al., 2012). In this line, Dijkstra and van Heuven (2002; BIA + model) proposed that language-detection mechanisms take place after lexical access has been completed, suggesting that it is not a basic initial stage of bilingual word processing. However, recent research has contradicted this view, demonstrating that bilinguals’ ability to use salient letter sequences in order to attribute the language of the words can help them speed up the word recognition process via the activation of the sub-lexical language nodes. At these early sub-lexical stages, orthographic markedness would help activating the correct language lexical system and partially inhibit cross-language lexical competitors. Hence, the presence of salient letter sequences reduces the amount of cross-language lexical interference during bilingual word reading (see Casaponsa et al., 2014; Casaponsa and Duñabeitia, 2016). The target word only competes with words within the language that have similar letter sequences, and this accelerates the decision on language attribution. This demonstrates that the orthographic (sub-lexical) language node is accessed before the lexical language node (see the BIA + s model proposed by Casaponsa et al., 2020).

Although adults are sensitive to markedness (Casaponsa et al., 2014), it is not clear whether this sensitivity is maintained throughout the lifespan or whether it is developed during a specific period of literacy consolidation. Previous research following the trajectory of biliteracy acquisition in bilingual children has shown that at initial stages of the development, word recognition heavily relies upon cross-language word similarity (see Duñabeitia et al., 2016). In this line, Duñabeitia et al. (2016) showed that cross-language lexical interactions in L1 and L2 word reading were reduced as the age of the readers increased. These results suggest that as bilinguals become more skilled readers, they rely less upon cross-language similarity in order to access the meaning of the words they read. Additionally, previous research has also shown that words that follow the phonotactic and orthotactic constraints of the native language are easier to learn and process (Bordag et al., 2017; Pérez-Serrano et al., under review). However, little is known about the role of orthographic distinctiveness across bilinguals’ two languages in relation to biliteracy acquisition. Presumably, bilingual children are able to detect sub-lexical language-specific patterns when reading, but the extent to which these patterns become cues that guide visual word recognition by speeding up language detection processes is yet to be explored.

The current study aims to examine how sensitive bilinguals are to markedness throughout childhood and early adulthood. The purpose is to examine the development of their ability to recognize marked (or unmarked) words from their languages at different ages, and to ascertain whether this ability changes or remains stable across life. In addition to allowing us to infer how sensitive people are to marked and unmarked bigrams, the current study also aims to replicate Casaponsa et al.’s (2014) findings with different age groups. If results vary with age, we can infer that children and adults differ in their ability to recognize sensitivity to marked words. Our results will show whether development during childhood changes how children detect language distinctiveness, as shown in previous experiments on implicit learning (Janacsek et al., 2012).

## Materials and Methods

### Participants

One hundred and twenty Spanish (L1) – Basque (L2) sequential bilinguals from the Basque Country participated in this experiment (77 females; age: *M* = 15.30, SD = 5.56, range: 8–29; age of L2 acquisition: *M* = 3.29, SD = 1.68). All participants received formal literacy instruction in Spanish and Basque simultaneously starting at the age of 6 years old (i.e., in Primary School), although exposure to Spanish and Basque printed materials already started in pre-school settings. It is worth noting that although Basque was formally acquired in the school context, the first contact with this language probably occurred at earlier stages, given that all participants were immersed in a bilingual society and their extended family members could either understand or speak Basque^1^). In order to facilitate the matching for critical variables, they were clustered according to their age into four groups of thirty participants each: children (17 females; *M*_age_ = 8.67 years, SD_age_ = 0.47), preteenagers (18 females; *M*_age_ = 12.40 years, SD_age_ = 0.62), teenagers (22 females; *M*_age_ = 16.97 years, SD_age_ = 0.31), and young adults (20 females; *M*_age_ = 23.01 years, SD_age_ = 2.74). All participants were right-handed, and none were diagnosed with language disorders, learning disabilities, or auditory impairments.

Adults were recruited from the University of the Basque Country, and the other three groups were recruited from a bilingual school. Adults, children, and children’s families were appropriately informed. Adult participants signed consent forms prior to the experiment. Parents or legal guardians signed the consent forms for underaged participants and also filled in a short language and socioeconomic status questionnaire before testing began. The protocol was carried out according to the guidelines approved by the BCBL Ethics and Scientific Committees. Adults were economically compensated, and the children were rewarded with a present.

We assessed all participants’ language proficiency, socioeconomic status, and IQ (see Table 1). Three measures were used to evaluate language proficiency. First, participants (or parents/guardians in the case of underaged participants) rated their language competence on a subjective scale from 0 to 10. Second, participants completed a lexical decision task (LexTale) in Spanish (Izura et al., 2014), Basque (de Bruin et al., 2017), and English (Lemhöfer and Broersma, 2012). Third, participants named twenty common objects from the adapted version of a picture naming task (de Bruin et al., 2017). In addition, we measured English proficiency. While this was not a relevant language for the task, we included this assessment in order to make sure that the participant’s English level was relatively low and would not have any effect on the other two languages (see Table 1). We also asked participants to state the percentage of time they were overall exposed to each language in a normal day to ensure similar language exposure across ages at the time of testing. Socioeconomic status was measured with a short questionnaire in which participants (or parents/guardians in the case of children) had to rate on a scale from 1 to 10 how they perceived their economic situation as compared to other members of their community (Adler and Stewart, 2007). Finally, IQ was measured with a 6-min abridged version of the K-BIT (Kaufman, 2004), in which participants had to complete as many matrices as they could in the allotted time.

**TABLE 1:** Descriptive statistics of demographic and language variables.

	**Children**	**Preteenagers**	**Teenagers**	**Adults**	**ANOVAs**	
					***F* (df)**	***p***
Age	8.67 (0.47)	12.40 (0.62)	16.97 (0.31)	23.01 (2.74)	*F*(3,116) = 55.98	<0.001
Age of L2 acquisition	3.40 (1.99)	3.50 (1.45)	3.30 (1.36)	2.97 (1.84)	*F*(3,116) = 0.56	0.639
Exposure to Spanish	62.67 (10.2)	63.00 (12.9)	62.67 (13.9)	60.83 (11.3)	*F*(3,116) = 0.19	0.899
Exposure to Basque	24.67 (7.64)	22.00 (7.83)	22.17 (8.97)	26.00 (11.7)	*F*(3,116) = 1.36	0.259
Exposure to English	12.67 (5.68)	15.00 (7.65)	15.17 (7.59)	13.17 (5.64)	*F*(3,116) = 1.07	0.364
Spanish competence	9.33 (0.75)	9.36 (0.71)	9.43 (0.72)	9.46 (0.73)	*F*(3,116) = 0.21	0.892
Basque competence	5.73 (1.61)	5.93 (0.98)	6.20 (1.44)	6.50 (1.67)	*F*(3,116) = 1.56	0.202
English competence	4.57 (1.63)	4.70 (1.51)	4.96 (1.21)	5.16 (1.48)	*F*(3,116) = 1.01	0.395
Spanish LexTale	69.36 (11.1)	87.69 (6.77)	92.97 (3.67)	93.05 (3.45)	*F*(3,116) = 77.01	<0.001
Basque LexTale	51.16 (11.9)	68.86 (10.2)	75.63 (13.2)	76.70 (12.3)	*F*(3,116) = 29.11	<0.002
English LexTale	52.08 (5.13)	53.87 (6.64)	57.29 (7.34)	56.45 (15.2)	*F*(3,116) = 1.92	0.13
Spanish picture naming	19.90 (0.30)	19.96 (0.18)	20.00 (0)	20.00 (0)	*F*(3,116) = 2.11	0.103
Basque picture naming	11.13 (2.83)	14.06 (3.62)	14.30 (4.47)	14.53 (4.81)	*F*(3,116) = 4.74	0.004
English picture naming	7.13 (3.97)	12.90 (3.38)	13.23 (4.31)	13.76 (3.80)	*F*(3,116) = 19.17	<0.001
Socioeconomic status	6.30 (1.29)	6.43 (1.67)	6.33 (1.34)	6.50 (1.10)	*F*(3,116) = 0.13	0.939
IQ	17.30 (2.15)	19.73 (2.39)	20.20 (2.65)	20.50 (2.94)	*F*(3,116) = 9.76	<0.001

*Values reported are means with standard deviation in parentheses for age (in years), age of acquisition (in years), language exposure (in % of exposed time), subjective language proficiency (0–10 scale), LexTale (average % of correct responses), picture naming (0–20 scale), economic status (1–10 scale), and IQ (number of correct answers). The last column shows the results from one-way ANOVAs comparing the four age groups on the different assessments.*

Participant groups were matched for their percentage of exposure to the three languages (Spanish, Basque, and English), their subjective language competence in the three languages, their Spanish picture naming skills, and their socioeconomic status (see Table 1). Different age groups could not be matched on the results of the lexical decision tasks (LexTale) or on IQ due to differences related to their development. [Note that vocabulary size increases with age thanks to exposure to new vocabulary (Hamilton et al., 2000), and that IQ also increases with age (Ramsden et al., 2013)].

#### Materials

##### Corpus of bigrams

A corpus of bigrams was compiled from Spanish (B-PAL; Davis and Perea, 2005) and Basque (E-HITZ; Perea et al., 2006) databases. First, diacritics and words containing letters that do not exist in one of the languages (ñ, c, q, v, w) were removed. All words were broken down into bigram units (e.g., the Spanish word for house, “*casa*,” was deconstructed as ca-as-sa). All bigram combinations were then averaged based on their appearance rates relative to all bigrams in terms of percentage (percentage frequency) in each the two languages. For example, the bigram *ca* appears in Spanish words 3482 times. The average number of appearances in the language is 1.57% (number of times a specific bigram appears × 100/total number of bigrams of that language).

##### Language decision task

In total, one hundred and sixty words were selected for the experiment. Half of the words were in Basque (selected from Perea et al., 2006) and the other half were in Spanish (taken from Davis and Perea, 2005). In both languages, two types of words were selected: marked and unmarked. Marked words contained one bigram that exists only in the target language and that is illegal in the other language. For example, “txakurra” – the Basque word for dog – is a marked word because the bigram “tx” does not exist in Spanish. We defined marked bigrams as those that had a frequency of use of 0 in the other language and a percentual bigram frequency of use higher than 0.1% in the target language. Following this rule, we selected four marked bigrams: two marked bigrams for Basque (“*tx*” and “*ts*”; percentual bigram frequency of use in Basque: 0.42 and 0.39%, respectively) and two for Spanish (“*mp*” and “*mb*”; percentual bigram frequency of use in Spanish: 0.31 and 0.28%, respectively). On the other hand, unmarked words contained only bigrams that exist in both languages and that have a high percentual bigram frequency of use (higher than 0.1%). For example, the bigram “rd” exists in both languages (as in “ardi,” the Basque word for sheep, and in “ardilla,” the Spanish word for squirrel) (see Appendix 1 to see the words used in the task).

Words were matched to control for the influence of classic characteristics that have been repeatedly shown to influence reading (see Table 2). First, we controlled for word length (in number of letters) and for word frequency of use, such that all words had a high frequency in the language (the frequency of use was bounded between 1 and 100 per million; see Table 2). Also, we matched the averaged percentual bigram frequency in each condition. We ensured that Spanish marked words had the same average bigram frequency in Spanish as Basque marked and unmarked words, so that none of the marked bigrams chosen was more salient in one of the languages. We also ensured that the bigrams had a high frequency of occurrence at each position within the word to avoid for potential positional confounds.

**TABLE 2:** Descriptive statistics of characteristics of the materials.

**Words**	**Spanish**		**Basque**	
	**Marked**	**Unmarked**	**Marked**	**Unmarked**
Word frequency (Zipf)	3.98 (0.67)	4.18 (0.29)	4.06 (0.59)	4.17 (0.58)
Word length	7 (1.43)	7 (1.46)	7 (1.45)	6.95 (1.35)
Spanish bigram frequency	0.71 (0.22)	0.72 (0.21)	0.53 (0.18)	0.69 (0.20)
Basque bigram frequency	0.52 (0.21)	0.69 (0.21)	0.71 (0.16)	0.72 (0.19)
Orthographic neighbors in Spanish	1.07 (1.43)	1.05 (1.31)	0.08 (0.22)	0.16 (1,49)
Orthographic neighbors in Basque	0.13 (1.43)	0.17 (1.08)	0.85 (1.31)	1.07 (1.28)
Length-corrected LD	0.14 (0.11)	0.12 (0.09)	0.13 (0.11)	0.13 (0.10)

**Pseudowords**	**Spanish marked**	**Basque marked**	**Unmarked**

Word length	7 (1.43)	7 (1.43)	7 (1.43)
Spanish bigram frequency	0.71 (0.16)	0.52 (0.17)	0.71 (0.23)
Basque bigram frequency	0.54 (0.18)	0.72 (0.16)	0.70 (0.26)
Orthographic neighbors in Spanish	0.1 (0.30)	0.02 (0.15)	0.22 (0.61)
Orthographic neighbors in Basque	0.02 (0,15)	0.42 (0.78)	0.12 (0.46)

*Values reported are means with standard deviation in parentheses for word frequency (Zipf scale), word length (number of letters), Spanish bigram frequency (percentage per million), Basque bigram frequency (percentage per million), orthographic neighbors (number of neighbors), and length-corrected LD (scale from 0 to 1).*

Given that the main task was to decide whether a given string corresponded to a Basque or a Spanish word, we also decided to control for the cross-linguistic overlap of the target items and their translations into the non-target language, in order to make sure that decisions could not be influenced by a high overlap between the items and their translation equivalents. To control for cross-linguistic similarity between the target word and its translation we controlled for the corrected orthographic Levenshtein distance. This measure accounts for the number of letters that differ between the translation equivalents. The length-corrected version of this measure ranges from a minimum value of 0, which refers to totally different translation equivalents, and 1, corresponding to completely overlapping cognates (e.g., the word piano in Spanish is the same word in English; Duñabeitia et al., 2013; Casaponsa et al., 2015). We wanted to avoid widespread overlap, so we picked words that had a maximum of 0.4 corrected Levenshtein distance (LD; see Table 2).

One-hundred sixty pseudowords were also created. Pseudowords were generated with Wuggy (Keuleers and Brysbaert, 2010) from the words described in the previous section. Pseudowords were added to the experiment because when participants process them, they have to base their answer on sub-lexical cues because there is no possible direct access to lexical or semantic information. Similar to the words, the pseudowords were also divided into Spanish marked, Basque marked, and unmarked pseudowords. Marked bigrams in pseudowords were the same as those used in the word set (“*tx*” and “*ts*” for Basque, and “*mp*” and “*mb*” for Spanish). The rest of the bigrams included in the marked pseudowords were unmarked bigrams that exist in both languages (see Appendix 2 for more examples). Unmarked pseudowords included only bigrams that exist in both languages. Given that unmarked words contained language-unspecific sub-lexical representations and lacked any lexical referent, they cannot be classified *a priori* as Spanish or Basque pseudowords.

#### Procedure

The whole experiment lasted about 30 min, including the language decision task and the language assessment. Participants were tested individually. Children were tested during school hours and adults during lab hours. All visual stimuli were presented on a computer with a 13-inch monitor running Experiment Builder^®^.

First, participants performed the language decision task. A fixation cross appeared on the center of the screen for 500 ms. Next, a word appeared until a response was given or for a maximum of 5000 ms. Participants were asked to respond as quickly as they could, indicating to which language (Basque or Spanish) each word belonged. They had to press the “C” key if the word belonged to Spanish or “B” if it belonged to Basque. In addition, participants were told that they would see pseudowords and that they had to decide which language each word could belong to. The order of presentation of the words and pseudowords was randomized for each participant.

#### Data Analysis

The dependent variables of interest collected in this experiment were Accuracy and Reaction Times (see Table 3). The R statistical environment (R Core Team, 2018) and Jamovi (The jamovi project, 2019) were used to analyze the data. Responses below 200 ms (considered as involuntary responses; 0.89% of the data) and timed out responses (1.04% of the data) were excluded from the analyses^2^. Moreover, erroneous responses were excluded from the latency analysis, and those responses three times the range interquartile above the third quartile or below the first quartile from the participant-based and item-based means in each condition were also discarded from the reaction time analysis (words: 3.31% of the data; pseudowords: 1.88% of the data). Response latencies and accuracy data were analyzed with linear and logistic mixed-effects models, respectively. Maximal models were fitted with random intercepts for participants and items and random slopes for all within-subject factors and their interactions. The random structure of the models was reduced when the data did not support the execution of the maximal model random structure in order to arrive at a parsimonious model. To do so, we computed principal component analyses (PCA) of the random structure (see Bates et al., 2015), and dropped the components that did not significantly contribute to the cumulative variance. Type-III ANOVA Wald-tests were computed to assess the significance of fixed effects for binary data using the *car* package, and Type-III ANOVA *F*-tests with Satterwhite approximations to degrees of freedom were computed for response latency analysis using the *lmerTest* package. In all models, the continuous predictor Age was scaled and centered prior to analyses. Categorical predictors were also centered by applying sum contrasts divided by the total number of levels of each factor.

**TABLE 3:** Descriptive statistics for the Language decision task.

	**Basque Words**		**Spanish Words**		**Marked Pseudowords**	
	**Marked**	**Unmarked**	**Marked**	**Unmarked**	**Basque**	**Spanish**
**Reaction times**						
Children	1736 (521)	1955 (591)	1831 (597)	1912 (590)	1882 (590)	2184 (683)
Preteenagers	1063 (249)	1199 (316)	1006 (206)	1061 (226)	1238 (340)	1663 (431)
Teenagers	810 (119)	935 (151)	782 (111)	821 (131)	959 (247)	1435 (414)
Adults	755 (115)	828 (129)	815 (106)	850 (124)	1000 (207)	1329 (360)
**Error rates**						
Children	14.12 (17.9)	23.98 (20.07)	18.2 (19.3)	19.0 (18.61)	15.4 (18.5)	53.03 (18.39)
Preteenagers	3.14 (4.17)	6.58 (6.72)	7.74 (9.28)	7.89 (8.68)	5.04 (7.94)	56.31 (21.84)
Teenagers	3.35 (4.18)	7.11 (6.58)	4.45 (4.54)	4.56 (4.31)	4.23 (4.26)	45.63 (24.6)
Adults	1.84 (2.3)	3.44 (3.65)	3.51 (3.56)	3.86 (2.9)	6.22 (5.47)	25.65 (16.42)

*Means with standard deviations in parentheses for accuracy rates (% errors) and reaction times (milliseconds) in each condition.*

The experiment design considered three main predictors. Language (Basque| Spanish) and Markedness (Marked| Unmarked) were considered as within-subject factors, and Age was considered a continuous variable. Words and pseudowords were analyzed separately, and unmarked pseudowords were analyzed based on the type of response choices, because unmarked pseudowords cannot be considered as correct or incorrect in terms of accuracy, since there are no language cues available to indicate to what language they belong. Hence, given that unmarked pseudowords were equally likely to be Basque-like or Spanish-like, they were analyzed separately. We report analyses of Type of Response (Basque| Spanish) as a function of Age for responses latencies on unmarked pseudowords. We also report analyses of language choice for unmarked pseudowords in order to identify whether participants displayed any potential bias toward a specific language on ambiguous strings, and how this might change as a function of age.

First, the word analysis was carried out. The percentage of correct responses and the reaction times for correct responses were analyzed including Language (Basque| Spanish) and Markedness (Marked| Unmarked) as within-subject factors, and Age as a continuous predictor. Second, the marked pseudowords were analyzed, including Language (Spanish-marked| Basque-marked) as a within-subject factor and Age as a continuous predictor. Third, response times on unmarked pseudowords were analyzed based on Response Type (Basque| Spanish) as a within-subject factor and Age as a continuous predictor. The probability of making a Basque choice for unmarked pseudowords was analyzed with the continuous predictor Age. Means and standard deviations of the reaction times and error rates in each critical condition are presented in Table 3 separated in four groups of age for the ease of interpretation.

## Results

### Words

**FIGURE 1:**
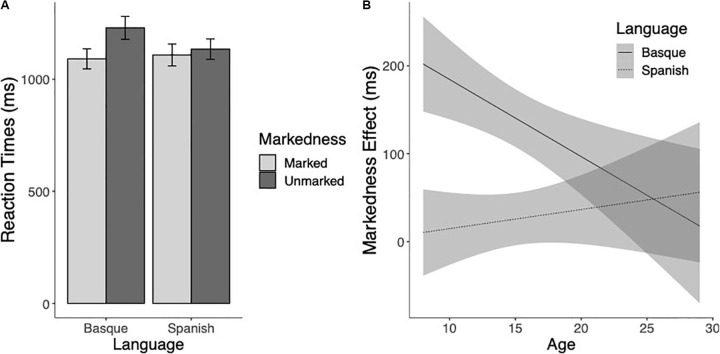
**(A)** Mean reaction times (in milliseconds) to marked and unmarked words for Basque and Spanish words. **(B)** Estimated marginal means of the linear regressions of the Markedness effect (unmarked minus marked) for Basque (thick line) and Spanish (dotted line) words as a function of age with the 95% confidence intervals.

The Reaction Time analysis showed a main effect of Markedness, so that marked words were responded to faster than unmarked words (*F*(1,170.7) = 38.51 *p* < 0.001). The Language effect was also significant, showing that responses to Basque words took on average longer than responses to Spanish words (*F*(1,196.7) = 6.74, *p* = 0.01). The effect of Age was also significant, demonstrating that RTs decreased with age (*F*(1,116.9) = 103.01, *p* < 0.001). Critically, the Markedness × Language × Age interaction was significant, showing that the markedness effect was different for Basque and Spanish, and that it was modulated by the age of the readers (*F*(1,110.78) = 8.79, *p* < 0.01). The markedness effect was present for Basque words (*t*(180.7) = −6.64, *p* < 0.001), but not for Spanish words (*t*(177.4) = −1.64, *p* = 0.10) (see Figure 1A). Furthermore, while the markedness effect was not modulated by the age of the participants for the Spanish words (*t*(114.2) = 0.17, *p* = 0.87), in the case of Basque words the magnitude of the markedness effect decreased with age (*t*(101.1) = −4.14, *p* < 0.001) (see Figure 1B).

**FIGURE 2:**
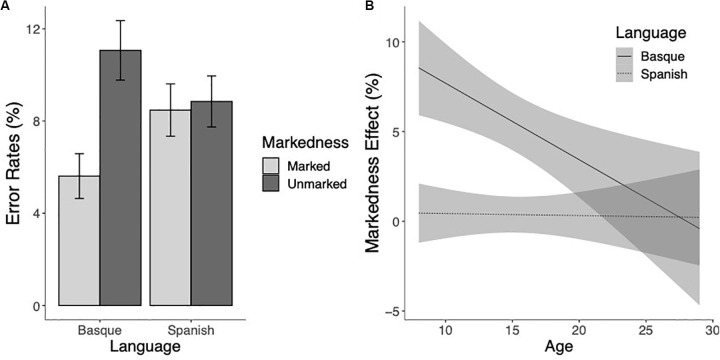
**(A)** Mean error rates (percentage of errors) to marked and unmarked words for Basque and Spanish words. **(B)** Estimated marginal means of the linear regressions of the Markedness effect (marked minus unmarked) for Basque (thick line) and Spanish (dotted line) as a function of age with the 95% confidence intervals.

The analysis of the error rates partially replicated these findings, showing a significant markedness effect, demonstrating that marked words elicited fewer errors than unmarked words (χ^2^(1) = 23.17, *p* < 0.001). The main effect of Language was not significant (χ^2^ = 1.79 and *p* = 0.18). The Age effect was significant, showing that accuracy increased with age (χ^2^(1) = 49.44, *p* < 0.001). Critically, the markedness effect interacted with language (χ^2^(1) = 44.44, *p* < 0.001). Pairwise comparisons confirmed that the markedness effect was present for Basque words (*z* = 7.62, *p* < 0.001), but not for Spanish words (*z* < 1, *p* > 0.70) (see Figure 2A). Although the magnitude of the markedness effect appeared to decrease with age for Basque words but not for Spanish words (see Figure 2B), the three-way interaction was not significant (χ^2^(1) < 1, *p* > 0.80).

### Marked Pseudowords

**FIGURE 3:**
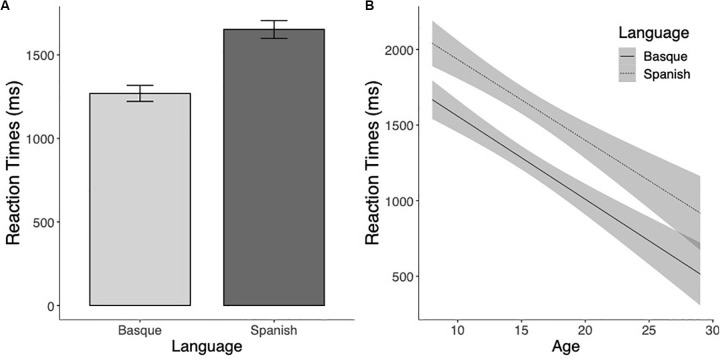
**(A)** Mean reaction times (in milliseconds) to Basque and Spanish marked pseudowords. **(B)** Estimated marginal means of the linear regressions of the effects of Language as a function of age with the 95% confidence intervals.

The analysis of the reaction times to marked pseudowords showed a significant effect of Language (*F*(1,145.8) = 15.23, *p* < 0.001), suggesting that pseudowords including Basque-specific letter combinations were identified faster than Spanish-like pseudowords (see Figure 3A). The Age effect was also significant (*F*(1,115) = 62.52, *p* < 0.001), showing that RTs decreased as a function of the age of the participants. The interaction between the two factors was not significant (*F* < 1 and *p* > 0.95, see Figure 3B).

**FIGURE 4:**
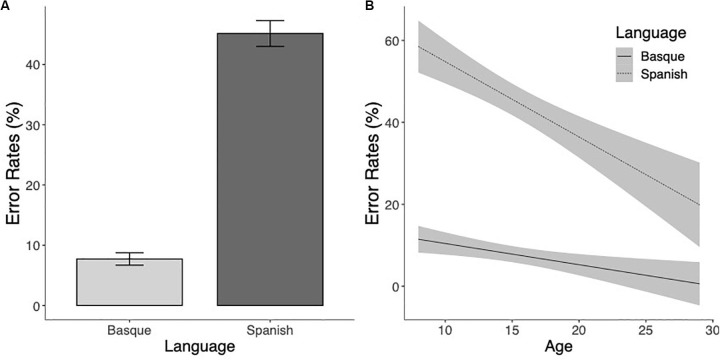
**(A)** Mean error rates (percentages of errors) to Basque and Spanish marked pseudowords. **(B)** Estimated marginal means of the linear regressions of the effects of Language as a function of age with the 95% confidence intervals.

A parallel analysis on the error rates also showed a significant effect of Language (χ^2^(1) = 135.88, *p* < 0.001), indicating higher percentages of errors for pseudowords with Spanish-specific bigrams than for pseudowords with Basque-specific bigrams (see Figure 4A). The Age effect was also significant (χ^2^(1) = 34.33, *p* < 0.001), showing that the error rates decreased as the age of the participants increased. The interaction between the two factors was not significant (χ^2^(1) = 1.59, *p* = 0.21), showing that error rates for both Basque (*z* = 2.35, *p* = 0.02) and Spanish (*z* = 5.23, *p* < 0.001) marked pseudowords decreased with Age (Figure 4B). In other words, the sensitivity to Basque-specific letter combinations and Spanish-specific letter chunks increased with age.

### Unmarked Pseudowords

**FIGURE 5:**
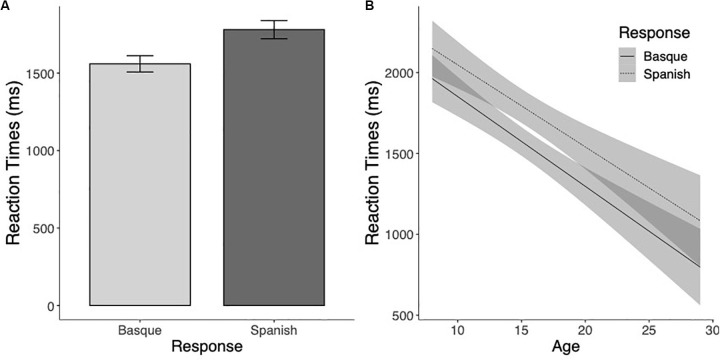
**(A)** Mean reaction times (in milliseconds) for Basque and Spanish choices on unmarked pseudowords. **(B)** Estimated marginal means of the linear regressions of the effect of Response type on unmarked pseudowords as a function of age with the 95% confidence intervals.

The analyses of reaction times to unmarked pseudowords as a function of type of response revealed that participants classified unknown and unmarked items as belonging to Basque faster than to Spanish (*F*(1,122.6) = 31.77, *p* < 0.001; see Figure 5A). The effect of age was also significant (*F*(1,116.9) = 37.32, *p* < 0.001), showing that response latencies decreased as age increased (see Figure 5B). These two factors did not interact (*F* < 1, *p* > 0.45).

**FIGURE 6:**
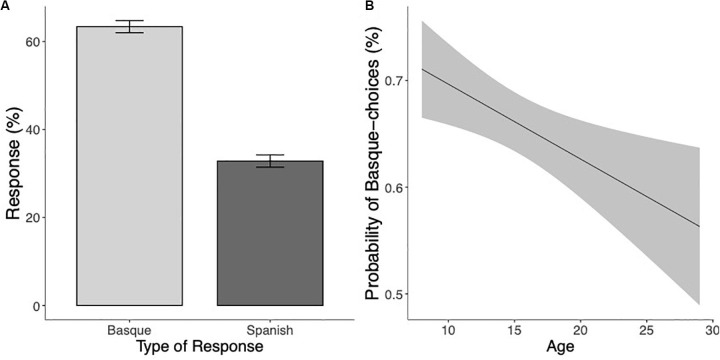
**(A)** Mean percentage of Basque and Spanish responses for unmarked pseudowords. **(B)** Estimated marginal means of a linear regression of the probability of Basque choices (in percentage) on unmarked pseudowords as a function of age with the 95% confidence intervals.

Participants’ bias toward Basque choices for ambiguous pseudowords significantly decreased as their age increased (χ^2^(1) = 7.94, *p* < 0.005), suggesting that at initial stages of bilingual literacy acquisition, participants attributed pseudowords lacking clear sub-lexical language cues primarily to their L2. This bias toward the less proficient language became less prominent as age increased (see Figure 6).

## Discussion

The aim of this study was to investigate whether sensitivity to markedness changed across the lifespan in bilinguals whose languages share the same alphabet but are orthotactically distinct. To this end, a large group of Spanish-Basque bilinguals whose ages were between 8 and 29 years was tested, including children, preteenagers, teenagers, and adults. Participants completed a language decision task with words and pseudowords that could include language-specific letter combinations. Results provided a better understanding of developmental stages, showing that sensitivity to markedness changed for the second language (Basque), while changes in the first language (Spanish) were limited to unknown words.

The current results showed that bilingual readers showed different sensitivity to markedness in their first and second language (in this study, Spanish and Basque, respectively). In the second language, people detected the language of the words more easily when they contained marked bigrams (e.g., “tx” is a marked bigram in Basque) than when they contained bigrams shared by the two languages. Similarly, when presented with pseudowords, readers detected the possible language with a significantly higher accuracy if the items included Basque-specific letter chunks than when they included Spanish-specific bigrams. This suggests that readers are highly sensitive to markedness in their second language, consistent with prior research (Lemhöfer and Dijkstra, 2004; Van Kesteren et al., 2012; Casaponsa et al., 2014; Chetail, 2015; Casaponsa and Duñabeitia, 2016).

In sharp contrast with the results obtained for items belonging to the non-native language (marked Basque words) or including bigrams that were specific to that language (Basque-marked pseudowords), readers showed minimal sensitivity to markedness in their native language. Participants performed equally well when presented with marked and unmarked Spanish words. These results suggest that readers might already be very good at detecting words in their native language and therefore the aid provided by native orthotactic cues is limited. Hence, in light of these results we can tentatively conclude that the importance of orthotactic cues is different depending on the knowledge of and experience with a language, being higher for non-native languages than for native ones.

We also examined how differences between sensitivity to markedness developed during childhood and adolescence. The current results showed that the degree of relevance of highly distinctive bigrams from the non-native language varied with age, and that their importance diminished as a function of age. In other words, while participants consistently identified words and pseudowords including bigrams that were Basque-specific (namely, Basque-marked items) significantly faster and more accurately than items containing Basque-unspecific letter combinations, this effect diminished as participants became older (see the Figures 1B, 2B; see also Table 3 for further insights). We tentatively interpret the finding of a smaller markedness effects as age increases as a result directly linked to augmented exposure to the print and enhanced biliteracy proficiency, similar to the findings observed with other markers of cross-language activation, such as the cognate effect (see Duñabeitia et al., 2016). As bilinguals become more skillful readers, words overall tend to be read faster and more accurately (see Table 3). The presence of orthographic cues based on markedness would still facilitate language attribution processes and word reading efficiency in the older participants, but the benefit might be less salient as compared to unmarked words due to the faster and more accurate reading of language ambiguous words, which would leave less room for facilitation effects to emerge.

In the current study we failed at finding any significant modulation of participants’ sensitivity to the bigrams that are specific to their native language as a function of their age for words that are known. Moreover, we did not find any signs of a markedness effect for the words of the native language, since responses to marked and unmarked Spanish words displayed similar accuracy rates and reaction times. These data could suggest that the sensitivity to the distributional properties of orthographic representations in the non-native language could be influenced by those of the native language, but that the opposite may not happen. That is, whilst L1 word processing does not seem to be markedly influenced by L2 orthotactics, L1 orthotactics have an impact on L2 reading. This aligns with the evidence from studies showing that second language learners normally display spillover or transfer effects from the native language. This malleability of the second but not first language led some authors to characterize the native language as stable and resistant, and the non-native one as weak and impressionable (e.g., Hernandez et al., 1994; Frenck-Mestre and Pynte, 1997). However, the lack of a facilitation effect for marked words in the native language and its steadiness across all ages might be masked by an advantage in word attribution and reading efficiency of Spanish words that are not marked. In other words, optimal processing of unmarked words in the native language during the different stages of biliteracy acquisition might result in a lower reliance on L1-specific sub-lexical cues.

It is worth noting that error rates for Spanish-marked pseudowords were modulated by age, revealing that the attribution to Spanish of Spanish-marked strings (i.e., pseudowords that violate L2 orthotactics) did increase over time. This thus suggests that as biliteracy skills develop, participants indeed become more sensitive to the intrinsic sub-lexical probabilities of their native language. These results are in line with previous research showing facilitation effects for L1-marked pseudowords in adult participants (see Oganian et al., 2015). Moreover, more recently, Borragán et al. (2020) also found facilitation effects for L1-marked pseudowords in older monolingual adults after learning a second language. The changes observed to the sensitivity to sub-lexical statistical regularities from the native language based on biliteracy acquisition found in the current study, aligns with more recent evidence showing that certain fundamental aspects of the first language can also change during the process of acquiring a second language (see, among many others, Baus et al., 2013; Kroll et al., 2015).

Note, however, that this conclusion might hold exclusively for the type of bilinguals tested in the current study. They were all early learners of the second language (with an age of second language acquisition around 3 years old) who were immersed in a bilingual society and exposed to the second language more than 20% of the time. Future studies should elucidate whether learning a new foreign language in a non-immersive scenario could yield different results. In a similar line, it should be noted that Basque and Spanish are languages with a shallow orthography, and it would be important to explore whether the same developmental effects also hold in languages with a deep (opaque) orthography, such as French or English. Previous research with skilled readers has already shown that combinations of languages with a deep orthography (e.g., French-English bilinguals) or combinations of languages with deep and shallow orthographies (e.g., Spanish-English bilinguals) is also influenced by the sensitivity to orthotactic cues (e.g., Vaid and Frenck-Mestre, 2002; Van Kesteren et al., 2012; Oganian et al., 2015; Casaponsa et al., 2020). Hence, in light of all the preceding evidence, we predict a similar pattern for the development of sensitivity to orthotactic cues in bilinguals who can read languages with different degrees of transparency in their orthographies, even though future studies will have to confirm whether this is indeed the case. Finally, future studies should explore whether orthographic markedness is also a factor that guides reading comprehension in more naturalistic reading scenarios that also involve sentence and text reading. We hypothesize that markedness effects will still occur in more naturalistic contexts, given that cross-language lexical competition has an impact across different reading comprehension scenarios (see Cop et al., 2017, for a study on book reading).

In sum, bilinguals whose languages are orthotactically different from each other are highly sensitive to the contrastive orthographic patterns of the second language, and they can use these orthotactic cues during reading (Casaponsa et al., 2014; Casaponsa and Duñabeitia, 2016). The main goal of this study was to investigate potential changes in the sensitivity to markedness across age, and thereby shed light on bilingual reading development to better characterize how the language of individual words is identified on the basis of the sub-lexical representations. These results suggest that bilingual readers are remarkably good at detecting orthotactic markedness in their non-native language, both when they have access to word meaning and when they do not (namely, with pseudowords), and this sensitivity changes as a function of age. In contrast, readers are only sensitive to orthotactic markedness in their native language when processing unknown words, and this sensitivity increases during biliteracy acquisition.

## Data Availability Statement

The raw data supporting the conclusions of this article will be made available by the authors, without undue reservation.

## Ethics Statement

The studies involving human participants were reviewed and approved by the Basque Center on Cognition, Brain and Language Ethics Committee. Written informed consent to participate in this study was provided by the participants’ legal guardian/next of kin.

## Author Contributions

MB, AB, and JD: conceptualization. MB, AC, and JD: data curation, formal analysis, and visualization. MB and JD: original draft preparation. MB, AC, AB, and JD: review and editing. All authors read and agreed to the published version of the manuscript.

## Conflict of Interest

The authors declare that the research was conducted in the absence of any commercial or financial relationships that could be construed as a potential conflict of interest.

## Footnotes

1 Spain is a country with multiple co-official or recognized languages in specific territories, such as in the Basque Country, where Basque and Spanish co-exist officially. Gipuzkoa, the region of the Basque Country where the study was carried out, is one of the regions were Basque is more prevalent (around 50% of the population are fluent Basque speakers and around an additional 15% are passive Basque-Spanish bilinguals).

2 Raw data can be retrieved from Duñabeitia et al. (2020, June 17). Changes in the sensitivity to language-specific orthographic patterns with age. Retrieved from osf.io/9r376 (https://osf.io/9r376/?view_only=861cad67c 4264e5d9ad07e7aa8d85a1a).

## Appendix 1. Words

**TABLE A1:** Words used in the experiment.

Basque		Spanish	
Marked	Unmarked	Marked	Unmarked
hauts (powder)	hodei (cloud)	tumba (tomb)	bruma (mist)
lotsa (shame)	ipuin (story)	bombo (drum)	plazo (time limit)
amets (dreams)	samur (tender)	rampa (ramp)	feliz (happy)
bitxi (jewel)	mutil (boy)	rumbo (course)	jaula (cage)
etsai (enemy)	hegal (wings)	bomba (bomb)	baile (dance)
txalo (applause)	afari (dinner)	ambos (both of them)	pelea (fight)
txano (cap)	sagar (apple)	impar (odd)	lunes (Monday)
untxi (rabbit)	ispilu (mirror)	pompa (pomp)	fibra (fiber)
otsail (february)	biloba (grandchild)	embudo (funnel)	abuelo (grandfather)
txistu (whistle)	epaile (judge)	mimbre (wicker)	dureza (hardness)
etxola (cabin)	aldapa (cost)	empate (tie)	pedazo (piece)
altxor (treasure)	amorru (rage)	limpio (cleansed)	regazo (lap)
txanda (turn)	abendu (december)	amplio (large)	hierba (grass)
txerto (vaccine)	bidaia (trip)	hambre (hungry)	huerto (orchard)
itxura (shape)	igande (sunday)	sombra (shadow)	espina (thorn)
itsaso (sea)	aginte (power)	nombre (first name)	guante (glove)
atsegin (pleasure)	egungo (current)	importe (amount)	humilde (humble)
txosten (memory)	jelosia (envy)	tumbona (deck chair)	enemigo (enemy)
atseden (break)	hedapen (expansion)	fiambre (cold meet)	rigidez (rigidity)
txantxa (joke)	sumendi (volcano)	siembra (sowing)	deporte (sport)
ahaltsu (powerful)	ostegun (thursday)	ombligo (belly button)	gigante (giant)
jatetxe (restaurant)	gauerdi (midnight)	tumbado (lying down)	semanal (weekly)
mingots (bitter)	langile (employees)	temblor (tremor)	resumen (summary)
etsipen (despair)	iraupen (duration)	asombro (astonishment)	soltero (single)
tximino (monkey)	egongela (room)	sombrero (hat)	usuario (user)
zoritxar (problems)	amildegi (cliff)	empinada (steep)	humildad (humility)
lainotsu (cloudy)	hiriburu (capital)	membrana (membrane)	paraguas (umbrella)
harritsu (rocky)	laburpen (summary)	temporal (temporary)	frialdad (coldness)
tximista (thunderbolt)	etorbide (avenue)	temprano (early)	detenido (deteined)
udaletxe (town hall)	omenaldi (tribute)	impuesto (tax)	heredero (inheritor)
gutxiegi (insufficient)	osotasun (integrity)	ambiente (ambient)	garganta (throat)
itsasalde (coast)	ibilaldi (walk)	empleado (employee)	plenitud (fullness)
itxaropen (hope)	argibide (instructions)	frambuesa (raspberry)	habilidad (ability)
lotsagabe (insolent)	gorespen (praise)	alumbrado (lighting)	peligroso (dangerous)
berdintsu (similary)	ondorengo (following)	ambulante (walking)	prometida (fiancee)
osasuntsu (healthy)	apaltasun (modesty)	tempestad (storm)	inestable (unstable)
gutxiengo (minority)	adeitasun (amiability)	imparable (unstoppable)	sobremesa (desktop)
tximeleta (butterfly)	lagunarte (company)	semblante (face)	resultado (result)
itsasadar (estuary)	iragarpen (prediction)	temporada (season)	siguiente (after)
igeltsero (builder)	abantaila (advantage)	limpiador (cleaner)	periodismo (journalism)

## Appendix 2. Pseudowords

**TABLE A2:** Pseudowords used in the experiment.

Basque			Spanish	
Marked	Unmarked		Marked	Unmarked
azots	sogen		dambu	falei
betsa	gamar		dempa	orbia
elets	ipola		ampes	antir
txisu	igore		ompal	aplur
lotsu	uduli		ombar	frola
butxa	esapi		ampel	nidru
netso	pangu		lampe	huiga
txosi	amapi		dombe	daulo
alitxo	gornen		tompal	igontu
betxor	onduri		grembo	filobe
bintxa	redain		bempon	jepola
atxela	pabrai		lambul	brafen
txinal	pigore		sampas	sofena
atxona	godupi		orambu	ugorel
txandu	olupen		alambo	repifo
daitsa	hurmar		lampir	oltala
etrutxo	harmile		simparu	ultorio
anditxo	blodatu		nambrol	nabalan
arotxun	enuarpe		empisor	errilta
lamatxa	lapurel		liamban	pugonel
ultatso	esmabra		lampuso	malurus
itsaton	erniepi		arombio	fablora
satxeta	neprisu		sarampo	dulaper
etxisan	dafaina		dasampo	luesmei
mirretxo	modurani		gestimbu	igergien
aistatxo	sadutelo		arrembon	manedari
berpitso	gegurone		darombas	prudarin
hungatso	palorego		onampegi	tomobrai
lutxandi	urmatino		anambolo	rusabrel
tsolasun	irubines		segampon	tagepiri
hastitxa	nestagun		eresombi	ilgarien
emotxeta	goruimon		saleompo	ruraiene
bitetxaba	ontapingo		pomboloti	ruirurpin
balutxeta	anirpento		arempobes	izomupero
lorintsol	surretimo		pimbredol	ramurdeta
aidetsolo	errudaimo		adempairo	darmongez
turontsus	saimanede		usimbento	rolganata
oraletsos	birrepudo		pampitros	femugeniz
eltoritso	badagaiso		ladarombe	gapifolas
putsielas	ruromolta		tampirela	osaumidar
